# Classification of Eye Fixation Related Potentials for Variable Stimulus Saliency

**DOI:** 10.3389/fnins.2016.00023

**Published:** 2016-02-15

**Authors:** Markus A. Wenzel, Jan-Eike Golenia, Benjamin Blankertz

**Affiliations:** Neurotechnology Group, Technische Universität BerlinBerlin, Germany

**Keywords:** EEG, eye tracking, eye fixation related potentials, search task, foveal vision, peripheral vision, saliency, single-trial classification

## Abstract

**Objective:** Electroencephalography (EEG) and eye tracking can possibly provide information about which items displayed on the screen are relevant for a person. Exploiting this implicit information promises to enhance various software applications. The specific problem addressed by the present study is that items shown in real applications are typically diverse. Accordingly, the saliency of information, which allows to discriminate between relevant and irrelevant items, varies. As a consequence, recognition can happen in foveal or in peripheral vision, i.e., either before or after the saccade to the item. Accordingly, neural processes related to recognition are expected to occur with a variable latency with respect to the eye movements. The aim was to investigate if relevance estimation based on EEG and eye tracking data is possible despite of the aforementioned variability.

**Approach:**Sixteen subjects performed a search task where the target saliency was varied while the EEG was recorded and the unrestrained eye movements were tracked. Based on the acquired data, it was estimated which of the items displayed were targets and which were distractors in the search task.

**Results:** Target prediction was possible also when the stimulus saliencies were mixed. Information contained in EEG and eye tracking data was found to be complementary and neural signals were captured despite of the unrestricted eye movements. The classification algorithm was able to cope with the experimentally induced variable timing of neural activity related to target recognition.

**Significance:** It was demonstrated how EEG and eye tracking data can provide implicit information about the relevance of items on the screen for potential use in online applications.

## 1. Introduction

Electroencephalography (EEG) and eye tracking can potentially be used to estimate which items displayed on the screen are relevant for the user. Exploiting this implicit information promises to enhance different types of applications and could, e.g., serve as additional input to computer software next to mouse and keyboard (cf. Hajimirza et al., 2012; Eugster et al., 2014, for the single modalities). Research on brain-computer interfacing (BCI) has shown that stimuli that are being paid attention to (targets) can be discriminated with EEG from other stimuli, that are not being attended to (distractors)—in certain experimental paradigms under laboratory conditions (Sutton et al., 1965; Farwell and Donchin, 1988; Picton, 1992; Polich, 2007; Treder et al., 2011; Acqualagna and Blankertz, 2013; Seoane et al., 2015). While BCI initially aimed at providing a communication channel for the paralyzed, recently non-medical applications gained increasing attention, such as mental state and cognitive workload monitoring (Blankertz et al., 2010), the control of media applications and games (Nijholt et al., 2009), cortically coupled computer vision for image search (Parra et al., 2008; Pohlmeyer et al., 2011; Ušćumlić et al., 2013), image categorization (Wang et al., 2012) and the detection of objects of interest in a three dimensional environment (Jangraw and Sajda, 2013; Jangraw et al., 2014).

In BCI experiments, stimuli are typically flashed on the screen and, therefore, the timing of stimulus recognition is precisely known. However, this information can not be expected in common software applications, where several possibly important items are displayed in parallel and not flashed in succession. In order to relate neural activity to the items on the screen, the eye movements can be tracked and the neural signals around the onsets of the eye fixations of the items can be inspected. Previously, EEG and eye tracking were measured in parallel to study eye-fixation-related potentials during reading (e.g., Baccino and Manunta, 2005; Dimigen et al., 2011, 2012) and search tasks (Sheinberg and Logothetis, 2001; Luo et al., 2009; Pohlmeyer et al., 2010, 2011; Rämä and Baccino, 2010; Dandekar et al., 2012; Kamienkowski et al., 2012; Brouwer et al., 2013; Dias et al., 2013; Kaunitz et al., 2014).

The present work is part of an endeavor that combines BCI technology with an application for interactive information retrieval (European project *MindSee*; www.mindsee.eu) for the improved exploration of new and yet unfamiliar topics (Glowacka et al., 2013; Ruotsalo et al., 2013). It builds on previous explorations to infer the cognitive states of users—such as attention, intent and relevance—from eye movement patterns, pupil size, electrophysiology and galvanic skin response with the objective to enhance information retrieval (Oliveira et al., 2009; Hardoon and Pasupa, 2010; Cole et al., 2011a,b; Gwizdka and Cole, 2011; Haji Mirza et al., 2011; Hajimirza et al., 2012; Kauppi et al., 2015).

Adding to the investigations published in the literature, the study presented here specifically addresses a problem resulting from a variable stimulus saliency. In real applications, it can be expected that the displayed items are diverse and that the saliency of target discriminative information is variable. Light entering the eye along the line of sight falls onto the fovea where the retina has the highest visual acuity. Peripheral retinal areas provide a lower spatial resolution (Wandell, 1995). Accordingly, relevant items can be detected either in foveal vision, or in peripheral vision—depending on the properties of the respective item and the attention of the participant. As a consequence, neural processes related to target recognition are expected to occur with a variable latency in relation to the eye movements (before or after the saccade to the item).

For the relevance estimation of single items displayed on the screen, it is required that a classification algorithm can detect EEG activity time locked to the onsets of the fixations of relevant items in single-trial. Hence, it was tested in the present study if this detection is possible even when the stimulus saliency is mixed, which leads to the aforementioned variability in terms of the neurophysiologic latency.

## 2. Materials and methods

### 2.1. Experimental design

**Figure 1:**
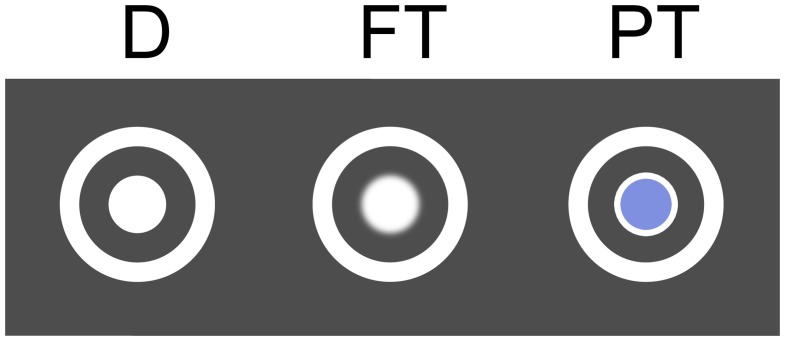
**Distractor (D), foveal target (FT), and peripheral target (PT)**.

The participants performed a gaze contingent search task while the electroencephalogram was recorded and the unrestrained eye movements were tracked. Twenty-four items situated at random positions on the screen had to be scanned and the number of targets among the distractors had to be reported. The saliency of target discriminative information was varied by using two types of targets, which could be recognized either in foveal vision or in peripheral vision. While distractors (D) featured a white disk (cf. Figure 1), foveal targets (FT) featured a white blurred disk and could be discriminated from distractors only in foveal vision. Accordingly, they had to be fixated for target detection (cf. Section 4.4). Peripheral targets (PT) featured a blue disk and could be discriminated from distractors already in peripheral vision. Fixations were not necessary for target detection (cf. Section 4.4) but were nevertheless required for task accomplishment (cf. last paragraph in this Section 2.1).

Among distractors, foveal targets were presented in the experimental condition **F** and peripheral targets in the condition **P**. The uncertainty that can be expected in realistic settings, where recognition can happen both in foveal and in peripheral vision, was modeled by the mixed condition **M**, where both types of targets were shown.

We rolled the dice for each of the 24 items displayed on the screen to decide if it is a target or a distractor (repeated for every repetition of the search task). Each item had the independent chance of being a target with a probability of 25% (allocated to 12.5% foveal and 12.5% peripheral targets in the mixed condition M). On average, there were 5.9 ± 2.2 (mean ± std) targets presented ranging from 1 to 12.

**Figure 2:**
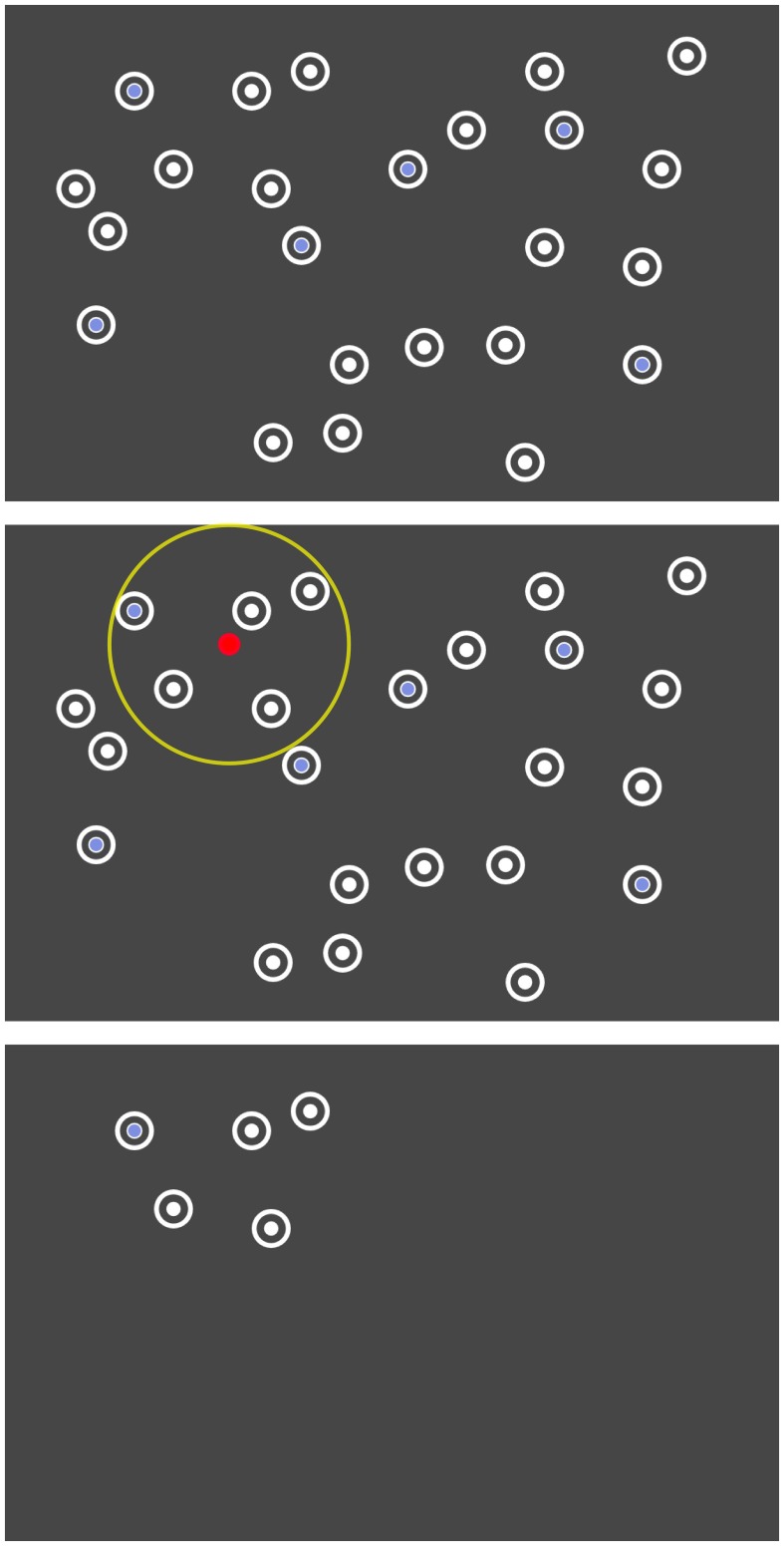
**Search task where the unrestrained gaze controlled the disclosure of the items. Top:** The arrangement of the items was predefined. **Center**: Only items within a certain radius (yellow) around the current point-of-gaze (red) were dynamically disclosed. **Bottom**: Illustration of the screen at one moment in the actual experiment.

The layout of the 24 items was predefined for each repetition of the search task (cf. last paragraph of this section). The items were initially hidden and were disclosed area by area, based on the eye gaze (cf. Figure 2). All items within a radius of 250 pixels (visual angle of 6.7°) around the current point-of-gaze were uncovered. When moving the gaze, previously hidden items appeared at the boundary of this circle. Thus, all items appeared in peripheral vision. The gaze contingent stimulus presentation was updated with 30 Hz based on the continuous eye tracker signal sampled with 250 Hz.

After leaving the radius of 250 pixel, the items disappeared again 1.5 s later. This gaze contingent disclosure allowed us to study event-related potentials (ERPs) aligned to the appearance of stimuli in peripheral vision and impeded the detection of all peripheral target items more or less at once by an unfocused “global” view on the whole screen.

Every item could disappear and reappear again in the gaze contingent stimulus presentation. However, as soon as an item was directly fixated (detected by the online algorithm of the eye tracker), it disappeared 1.5 s later and did not reappear again. Note that it was not necessary to fixate the item for 1.5 s. This behavior forced the participants to discriminate between targets and distractors upon the first fixation of an item and impeded the careless gaze on items, which would probably attenuate components of the ERP that are related to target recognition.

The three conditions of the search task were repeated 100 times each resulting in 300 repetitions in total. Before the beginning of each repetition of the search task, a fixation cross had to be fixated until it disappeared after 2 s. As soon as all target items had been fixated, the stimulus presentation ended and the question to enter the number of targets was addressed. Finally, the participant was informed if the answer was correct or not by a “happy” or a “sad” picture to enhance task engagement. Ten subsequent repetitions of one condition built one block. The blocks of the three conditions were interleaved and the participants were informed about the respective condition at the beginning of each block.

### 2.2. Experimental setup

The participants were seated in front of a screen at a viewing distance of sixty centimeters and entered the counted number of targets with a computer keyboard. An eye tracker (*RED 250*, SensoMotoric Instruments, Teltow, Germany; sampling frequency of 250 Hz) was attached to the screen and a chin rest gave orientation for a stable position of the head. The gaze contingent stimulus presentation was updated with 30 Hz. The screen itself had a refresh rate of 60 Hz, a resolution of 1680 × 1050 pixels, a size of 47.2 × 29.6 cm and subtended a visual angle of 38.2° in horizontal and of 26.3° in vertical direction. The target and distractor items had a diameter of 50 pixels, subtended a visual angle of 1.3°, and had a minimal distance of 70 pixels or 1.9° between each other and of 100 pixels or 2.7° from the border of the screen. An item was considered as fixated if the fixation position was situated within a radius of 75 pixels or 2.0° from the center of the item and no other item was closer.

Physiological signals were recorded with 64 active EEG electrodes including one electrode situated below the left eye for electrooculography (*BrainAmp, ActiCap*, BrainProducts, Munich, Germany; sampling frequency of 1000 Hz). The ground electrode was placed on the forehead, the reference electrode on the left mastoid and one of the regular EEG electrodes on the right mastoid for later re-referencing (see Section 2.3). The vertical electrooculogram (EOG) was computed by subtracting the electrode Fp1 from the electrode below the left eye. The horizontal EOG was yielded by subtracting the electrode F9 from the electrode F10.

**Figure 3:**
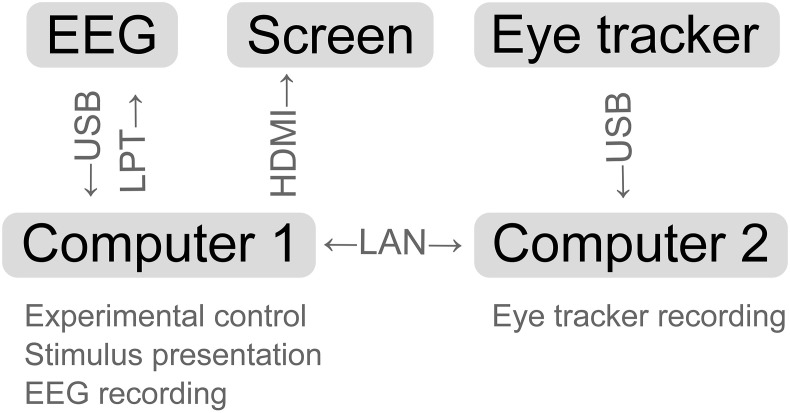
**Schematic representation of the experimental setup**. Arrows indicate the data flow between the devices.

To accomplish the dynamic stimulus presentation and multi-modal data acquisition, *Matlab* and *Python* code was written. The following software programs were running on two computers and interacting: *Pyff* for stimulus presentation (Venthur et al., 2010), *BrainVisionRecorder* (BrainProducts, Munich, Germany) for EEG data acquisition, *iView X* (SensoMotoric Instruments, Teltow, Germany) for eye tracking and online fixation detection and the *iView X API* to allow for communication between the computers (see Figure 3 for a schematic representation).

### 2.3. Data acquisition

Sixteen persons with normal or corrected to normal vision and no report of eye or neurological diseases participated in the experiments. The age of the four women and twelve men ranged from 18 to 54 years and was on average 30.7 years. One recording session included giving an informed written consent to take part in the study, vision tests for visual acuity and eye dominance, preparation of the sensors, eye tracker calibration and validation, introduction to the task and to the gaze contingent stimulus presentation, training runs, the main experiment (with a duration of about 2 h), and standard EEG measurements (eyes-open/closed, simple oddball paradigm, see Duncan et al., 2009). The proper calibration of the eye tracker was re-validated and—if necessary—re-calibrated in the middle of the experiment and in the case that the subject reported that the items did not disappear after fixation. The study was approved by the ethics committee of the Department of Psychology and Ergonomics of the Technische Universität Berlin.

The synchronously recorded EEG and eye tracking data were aligned with the help of the sync triggers, which had been send via parallel port interface (LPT) to the EEG system every second during the experiment, and the time-stamps of the eye tracker logged at the same time. The parameters of the function mapping eye-tracking-time to EEG-time were determined with linear regression. The EEG data were low-pass filtered with a second order Chebyshev filter (42 Hz passband, 49 Hz stopband), down-sampled to 100 Hz, re-referenced to the linked-mastoids and high-pass filtered with a FIR filter at 0.1 Hz.

For the EEG analysis, fixation-onsets were determined from the (continuous) eye tracker signal sampled at 250 Hz with the software of the eye tracker (*IDF Event Detector*, SensoMotoric Instruments, Teltow, Germany; event detection: “high speed,” peak velocity threshold: 40°/s, min. fixation duration: 50 ms) and the first fixations onto target and distractor items were selected.

### 2.4. Data analysis

#### 2.4.1. Search task performance

The performance of the participants in the search task was assessed with the percentage of correct responses and with the absolute differences between response and true number of targets. It was tested whether the experimental conditions differed in these respects with one-way repeated measures analyses of variance.

#### 2.4.2. Target estimation with EEG and eye tracking features

Based on EEG and eye tracking data, it was estimated which items displayed on the screen were targets, and accordingly relevant for the person to solve the search task, and which were distractors. For this purpose, feature vectors were classified, which had been extracted from EEG and eye tracking data. Each feature vector was either labeled as *target* or as *distractor* depending on the corresponding item.

##### 2.4.2.1. EEG features.

The continuous multichannel EEG time-series were segmented in epochs of 0 ms to 800 ms relative to the onset of the first fixation of each item. Each epoch was channel-wise baseline corrected by subtracting the mean signal within the 200 ms before the fixation-onset. The EEG signal measured at each channel was then averaged over 50 ms long intervals and the resulting mean values of all channels and all intervals were concatenated in one feature vector per epoch (that represents the spatio-temporal pattern of the neural processes). Improved classification performance is intended goal of this step-via a reduction of the dimensionality of the feature vectors in comparison to the number of samples (cf. the Section “Features of ERP classification” in Blankertz et al., 2011).

##### 2.4.2.2. Eye tracking features.

From the eye tracking data, the duration of the first fixation of each item and the duration and distance of the respective previous and following saccade were determined and used as features.

EEG and eye tracking features were classified both separately (“EEG,” “ET”) and together (“EEG and ET”)—by appending the eye tracking features to the corresponding EEG feature vectors—with regularized linear discriminant analysis. The shrinkage parameter was calculated with an analytic method (see Friedman, 1989; Ledoit and Wolf, 2004; Schäfer and Strimmer, 2005, for more details). More information about this approach to single-trial ERP classification is provided in Blankertz et al. (2011). The classification performance was evaluated in 10 × 10-fold cross-validations with the area under the curve (AUC) of the receiver-operator characteristic, which is applicable for imbalanced data sets (more distractors than targets; Fawcett, 2006). The better the classification performance, the more the AUC differs from 0.5. The classifications were performed separately for each combination of participant, experimental condition (F, P, M) and modality (“EEG,” “ET,” “EEG and ET”). Per condition and modality, it was assessed with one-tailed Wilcoxon signed-rank tests whether the median classification performance of all participants was significantly better than the chance level of an AUC of 0.5.

##### 2.4.2.3. Electrooculogram.

The classifications were additionally performed using only the horizontal and the vertical electrooculogram (“EOG”). The same feature extraction method was employed for the EOG channels as for the EEG. The aim was not to get the best possible classification from the EOG, but to check whether the performance of the EEG-based classification is in part based on EOG signals and, therefore, can be explained to a certain extent by eye movements as confounding factor.

Subsequently, it was tested with a two-way repeated measures analysis of variance, if the experimental conditions (F, P, M) and the modalities (“EEG,” “ET,” “EEG and ET,” “EOG”) had an effect on the classification performance.

Two additional analyses of the EEG data of the mixed condition M were conducted, where there were both peripheral and foveal targets present as well as distractors:

- A *combined classifier* consisting of a combination of two classifiers was designed. One classifier was trained to discriminate foveal targets from distractors and another classifier learned to discriminate peripheral targets from distractors—both using fixation-aligned EEG epochs from condition M. The two classifiers were then applied to the respective test-subset of a 10 × 10 crossvalidation, where the saliency of the target items (foveal or peripheral) was not unveiled. The posterior probabilities yielded from the two classifiers were averaged for each EEG epoch to predict if it was a target or a distractor epoch (Tulyakov et al., 2008). It was tested if the combined classifier was better able than the standard classifier to cope with the temporal variability of the neural response in relation to the eye movements, which was present in the mixed condition M and which can be expected in realistic settings.
- A reference case for the achievable classification performance would be represented by a *split analysis*, where peripheral and foveal targets are treated separately. This models a situation (which usually can not be expected in the application case) where the saliency of each item is known and, accordingly, whether the item can be recognized in peripheral vision or not. For this purpose, the EEG data of the mixed condition M were split and either foveal or peripheral targets were classified against distractors using fixation-aligned EEG epochs. The distractor data were split arbitrarily in halves.

##### 2.4.2.4. Appearance-aligned EEG features.

Furthermore, it was tested if information was present in the EEG data already when the items appeared in peripheral vision, i.e., even before fixation-onset (cf. the description of the gaze-contingent stimulus presentation in Section 2.1). For this purpose, the EEG time-series were segmented in epochs aligned to the first appearance of each item on the screen. Baseline correction of the 800 ms long epochs was performed using the 200 ms interval before the appearance. Features were extracted and classified as described above for the fixation-aligned EEG epochs.

#### 2.4.3. Characteristics of target and distractor EEG epochs

The EEG data were further characterized to provide insights into the underlying reasons for success or failure of the classifications and into the neural correlates of peripheral and foveal target recognition.

##### 2.4.3.1. EEG epochs aligned to item appearance and fixation.

The EEG time-series were segmented in epochs aligned to the first appearance of each item on the screen (caused by gaze movements, cf. Section 2.1) and in epochs aligned to the first fixations of the items (cf. Section 2.4.2). Each 1000 ms long epoch started 200 ms before the appearance or fixation, was channel-wise baseline corrected by subtracting the mean signal within the 200 ms interval before the respective event and was labeled as *target* if the corresponding item was a target and otherwise as *distractor*.

##### 2.4.3.2. Class-wise averages.

Single EEG epochs contain a superposition of different components of brain activity, including non-phase locked oscillatory signals. Averaging the EEG epochs attenuates the non-phase locked components. The average is referred to as the event-related potential, which is abbreviated as ERP. To single out the phase locked brain activity, target, and distractor EEG epochs of all participants were class-wise averaged. The two types of events (appearance, fixation-onset) and the three experimental conditions (F, P, and M) were assessed separately. Before averaging, artifacts were rejected with a heuristic: channels with a comparably small variance were removed as well as epochs with a comparably large variance or with an absolute signal amplitude difference that exceeded 150 μV (only the interval of 800 ms after the appearance or fixation was considered for artifact rejection). Artifact rejection was used for the visualization in order to obtain clean ERPs. For single-trial classification we preferred to take on the challenge of dealing with trials that are corrupted by artifacts as this is beneficial for online operation in future use cases. Due to the usage of data-driven multivariate methods, many types of artifacts can indeed be successfully projected out. The influence of eye movements on the EEG data are discussed in the Sections 4.2 and 4.5.

##### 2.4.3.3. Statistical differences between classes.

Target and distractor EEG epochs were compared with univariate statistics. Differences between the epochs of the two classes were quantified per subject, for each channel, and each time point with the signed squared biserial correlation coefficient (signed *r*^2^) between each univariate feature and the class label (+1 for targets and −1 for distractors). A signed *r*^2^ of zero indicates that feature and class label are not correlated and a positive value indicates that the feature was larger for targets than for distractors and vice versa. In an across-subject analysis, the individual coefficients were aggregated into one grand average value for each univariate feature. The *p*-value related to the null hypothesis that the signed *r*^2^ across all subjects is zero was derived.

##### 2.4.3.4. Classifications with either spatial or temporal EEG features.

While spatio-temporal EEG features served for the actual classification purpose (cf. Section 2.4.2), the classification with either temporal features or spatial EEG features allowed to specify where the discriminative information resided in space and time (see Blankertz et al., 2011). In the case of temporal features, the time-series were classified separately for each EEG channel, using the interval of 800 ms post-event. The AUC-scores obtained for each channel were averaged over participants and displayed as scalp maps. In the case of spatial features, the EEG epochs were split in 50 ms long (multi-channel) chunks, which were averaged along time. The resulting feature vectors were classified separately for each chunk and the mean AUC-scores of all participants were displayed as time courses.

#### 2.4.4. Eye gaze characteristics

The eye movements of the participants were characterized with the average fixation duration of each item type in each experimental condition. In addition, the fixation frequency was computed, i.e., the number of the fixations on each item type in comparison to the total number of fixations on all item types. Besides, the average duration and distance of the first saccades to the items and of the respective following saccades were calculated. Moreover, the average latency between the first appearance of each item and its fixation were determined. Re-fixations of items were not considered because, then, the identity of the item had been already revealed.

## 3. Results

### 3.1. Search task performance

The participants gave correct responses in condition F in 70.6 %, in P in 81.3 %, and in M in 75.1 % of the cases. The absolute differences between response and true number of targets were 0.370 in F, 0.255 in P, and 0.350 in M. These two performance measures differed significantly between conditions [one-way repeated measures analyses of variance, *F*_(2, 30)_ = 11.7, *p* ≤ 0.01 and *F*_(2, 30)_ = 5.88, *p* ≤ 0.01].

### 3.2. Target estimation with EEG and eye tracking features

It was estimated which items displayed on the screen were targets of the search task based on EEG and eye tracking data. The results of the classifications are listed in Table 1. The two modalities were either classified together (“EEG and ET”) or separately (“EEG,” “ET”). Additionally, features only from the electrooculogram (“EOG”) were used to investigate to which degree eye movements might have confounded the classifications with EEG features. The classification performance was better than chance in all experimental conditions and for all modalities except for the EOG features (one-tailed Wilcoxon signed-rank tests, *p* ≤ 0.01, Bonferroni corrected for the 12 comparisons).

**Table 1:** **Classification results are listed for the different modalities and the three experimental conditions**.

	**F**	**P**	**M**
EEG and ET	0.726^*^ ± 0.054	0.714^*^ ± 0.070	0.678^*^ ± 0.060
EEG	0.672^*^ ± 0.060	0.633^*^ ± 0.055	0.620^*^ ± 0.047
ET	0.677^*^ ± 0.044	0.718^*^ ± 0.084	0.652^*^ ± 0.061
EOG	0.516 ± 0.020	0.536 ± 0.033	0.514 ± 0.020

“*EEG and ET” denotes the multimodal classification of EEG and eye tracking features, “EEG” and “ET” stand for the separate assessments with either one or the other modality and “EOG” for the classifications with features from the electrooculogram only. In the table, AUC-scores are presented as averages over participants together with the corresponding standard deviations. Asterisks mark results significantly above the chance level 0.5 (one-tailed Wilcoxon signed-rank tests, p ≤ 0.01, Bonferroni corrected for the 12 comparisons).*

The modalities as well as the experimental conditions had a significant effect on the classification performance [two-way repeated measures analysis of variance, *F*_(3, 165)_ = 203, *p* ≤ 0.01 and *F*_(2, 165)_ = 14.6, *p* ≤ 0.01].

Using EEG and eye tracking features in combination resulted in classification performances that were significantly better than when either eye tracking or EEG features were used alone. Significantly better results were obtained with eye tracking features than with EEG features (one-tailed Wilcoxon signed-rank tests, *p* ≤ 0.01, Bonferroni corrected for the three comparisons). The individual classification performances ranged from 0.556 to 0.828 in the case of “EEG and ET,” from 0.529 to 0.765 in the case of “EEG,” and from 0.543 to 0.862 in the case of “ET” (averages and standard deviations are listed per condition in Table 1). The individual results for “EEG” and “ET” did not correlate significantly (*p* > 0.01).

The ranking of the three experimental conditions according to the classification performance was F > P > M in the case of “EEG and ET” and “EEG” and P > F > M in the case of “ET” (cf. Table 1). The classification performance was significantly better in condition F than in condition M in the cases of “EEG and ET” and of “EEG” and significantly better in P than in M in the case of “ET” (one-tailed Wilcoxon signed-rank tests, *p* ≤ 0.01, Bonferroni corrected for the three comparisons).

Per participant, condition and modality, about 544 target vs. 1181 distractor samples were, on average, available for the classification. These numbers result from the about 24^*^0.25^*^100 = 600 targets and 24^*^0.75^*^100 = 1800 distractors presented in total and the fact that not all items were fixated by the participant (cf. Section 3.4 and Table 3).

The results of the two additional analyses of the fixation-aligned EEG epochs from condition M are listed in Table 2. For the *combined classifier*, one classifier had been trained to discriminate foveal targets from distractors and a second classifier to discriminate peripheral targets from distractors. Both classifiers were applied to the test data in combination by averaging the posterior probabilities yielded per epoch. The combined classifier performed, on average, slightly better than the standard EEG-based classifier (cf. Table 1, row “EEG,” column “M”), however not significantly (*p* > 0.01). In the *split analysis*, either foveal or peripheral targets were classified against distractors. The performance of the classification of foveal targets vs. distractors (“FT vs. D”) was significantly better than the standard EEG-based classification of condition M (*p* ≤ 0.01) and comparable to the result of condition F (cf. Table 1, row “EEG,” columns “M” and “F”).

**Table 2:** **Results of the combined classifier and the split analysis in condition M (averages and standard deviations of the 16 participants of the study)**.

**Method**	**Classes**	**[AUC]**
Combined classifier	PT, FT vs. D	0.627 ± 0.042
Split analysis	FT vs. D_1∕2_	0.666 ± 0.065
Split analysis	PT vs. D_2∕2_	0.590 ± 0.036

All classification results were significantly above the chance level of an AUC of 0.5 (one-tailed Wilcoxon signed-rank tests, p ≤ 0.01, Bonferroni corrected for the three comparisons). For the split analyses, the distractor data were split arbitrarily in halves (denoted as D_1/2_ and D_2/2_).

#### 3.2.1. Appearance-aligned EEG features.

Classification performance was better in condition P than in the conditions F and M (F: 0.518 ± 0.018, P: 0.637 ± 0.044, M: 0.547 ± 0.023). In all conditions, the performance was significantly better than the chance level (one-tailed Wilcoxon signed-rank tests, *p* ≤ 0.01, Bonferroni corrected for the three comparisons).

### 3.3. Characteristics of target and distractor EEG epochs

#### 3.3.1. Class-wise averages

**Figure 4:**
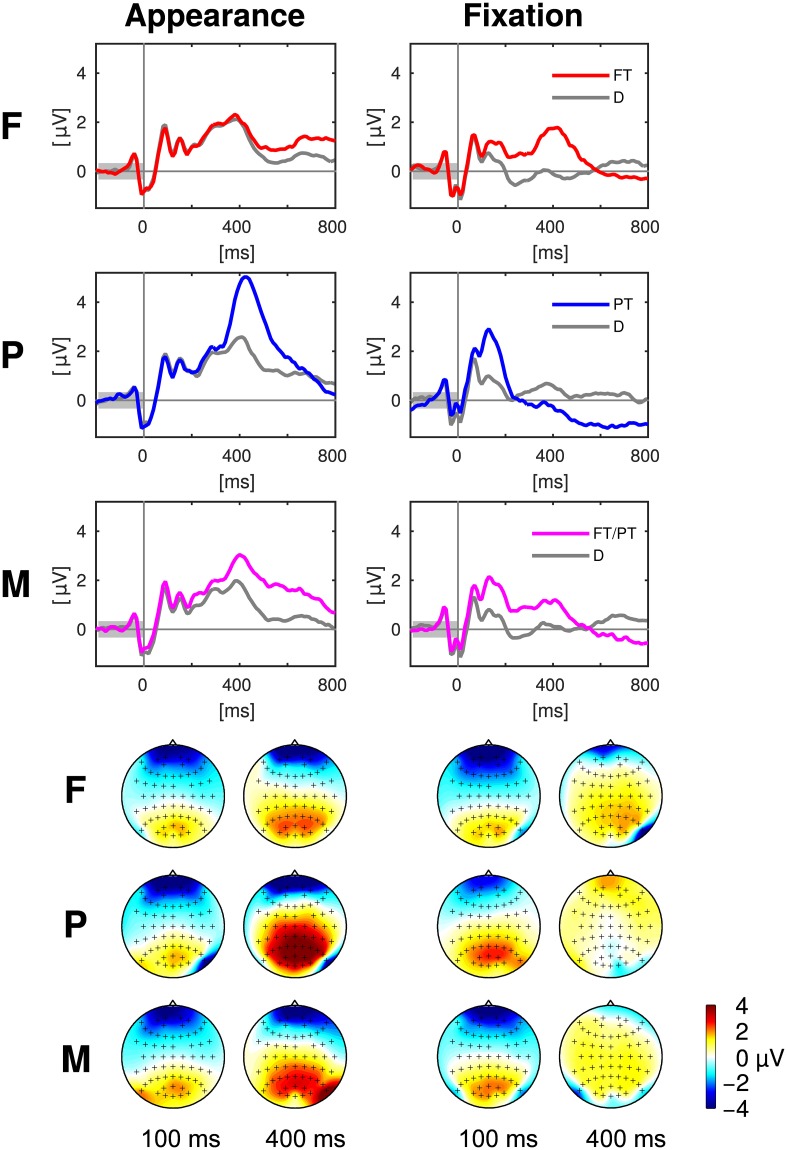
**Top:** Event related potentials aligned to appearance and fixation of targets (colored) and distractors (gray) at the exemplary electrode Pz in the experimental conditions F, P, and M. **Bottom:** The scalp maps depict the head from above with the nose on top and show the potentials averaged over 50 ms long intervals centered at 100 and 400 ms after the target appearance (left) and fixation (right). Please note that the positivity (“yellow/orange/red”) at central, parietal and occipital electrodes was discriminative between targets and distractors in contrast to the negativity (“blue”) at prefrontal and anterior frontal electrodes (cf. Figure 5). Every figure throughout the paper summarizes the data of all 16 participants of the study.

**Figure 5:**
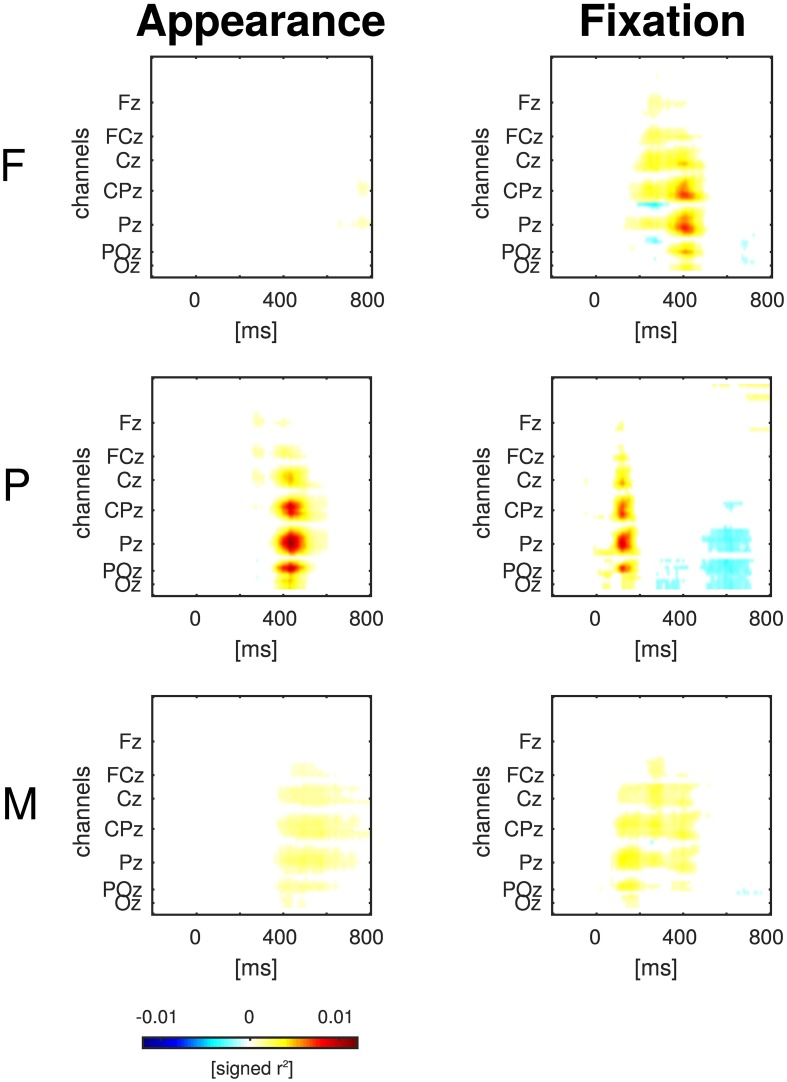
**Statistical differences between target and distractor EEG epochs aligned to item appearance and fixation in the conditions F, P, and M (across-subject signed *r*^2^-values)**. The channels are ordered from the front to the back of the head (top to bottom in the figure).

The class-wise averages of the EEG epochs are presented in Figure 4. The two types of events (*appearance* of an item on the screen and onset of the eye *fixation*, cf. Section 2.4.6) and the three experimental conditions (F, P, M) were assessed separately. Electrode Pz was chosen for the presentation as time course, because it is well suited to capture the P300 wave (Picton, 1992). Note that information regarding all electrodes and all time points is presented in the next Section 3.3.2 with Figure 5.

#### 3.3.2. Statistical differences between classes

The statistical differences between target and distractor EEG epochs aligned either to item appearance or fixation are shown in Figure 5. Significant differences (*p* ≤ 0.01, Bonferroni correction for multiple comparisons due to the number of channels, time-points, conditions and event types) occurred mainly at central, parietal and occipital electrodes close to the mid-line of the head. Across-subject signed *r*^2^-values that were not significantly different from zero were set to zero and remain white in the figure.

#### 3.3.3. Classifications with either spatial or temporal EEG features

**Figure 6:**
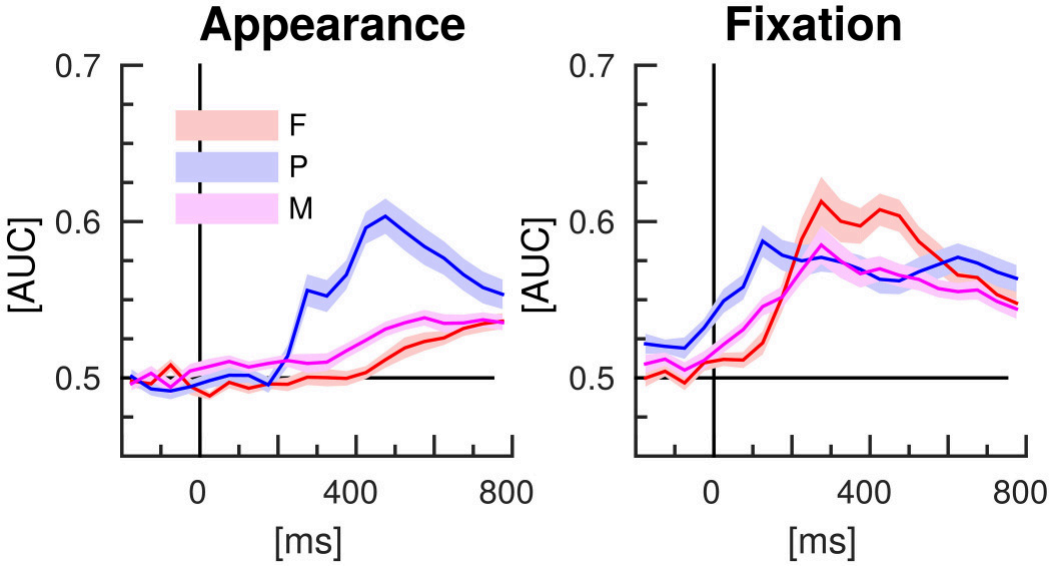
**EEG classification with spatial features (separate time-intervals at all channels)**. Lines indicate the mean AUC-scores of the 16 participants of the study and shaded areas stand for the standard error of the mean.

**Figure 7:**
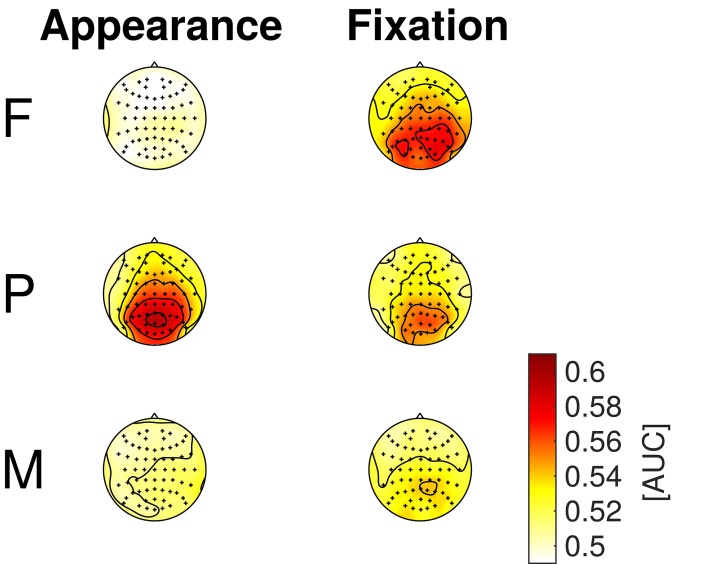
**EEG classification with temporal features (entire time-series of separate channels)**. Average AUC-scores of the 16 participants are presented in color code as scalp maps.

The results of the classifications of target vs. distractor EEG epochs, using either spatial or temporal features are presented in the Figures 6, 7, respectively. The EEG epochs were aligned either to item appearance or fixation. The three experimental conditions F, P, and M were assessed separately.

*Spatial EEG features* (i.e., data from separate time-intervals at all channels) of target vs. distractor epochs were classified to characterize where the information resided in time. Figure 6 depicts the time courses of the classification performance averaged over subjects. In condition P, classification performance started to surpass the chance level of an AUC of 0.5 at about 200 ms after item appearance and reached the maximum at about 500 ms post-appearance with an AUC of about 0.6. In the conditions F and M, only a slight increase over time was observed after item appearance. In contrast, classification performance increased clearly in all three conditions in response to the fixation-onset. The maximum was reached faster in condition P, at about 150 ms, than in the conditions F and M, at about 300 ms. In condition P, the AUC values exceeded the chance level even before fixation-onset.

*Temporal EEG features* (i.e., the entire time-series of separate channels) were used to classify between target and distractor epochs to learn where the discriminative information resided in space. Figure 7 depicts the classification results as scalp maps (AUC-scores for each channel, averaged over participants). Channels situated at central, parietal, and occipital positions showed the largest AUC-values and were, accordingly, most informative about the class membership.

### 3.4. Eye gaze characteristics

**Figure 8:**
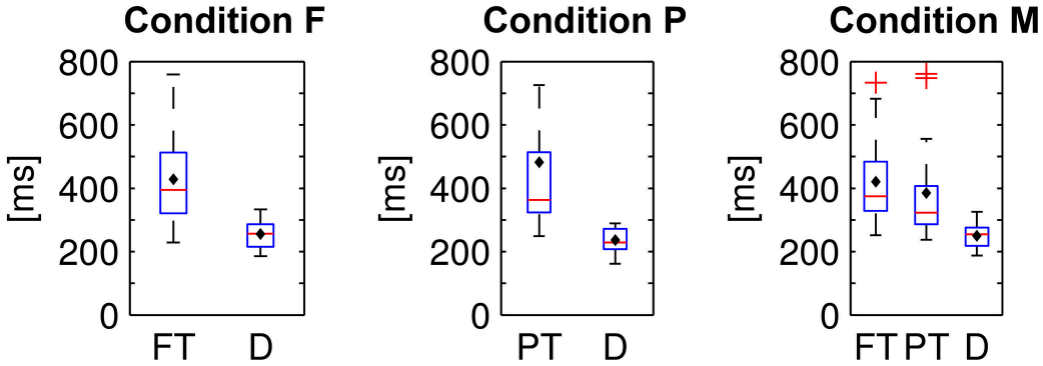
**Fixation durations of foveal (FT) and peripheral targets (PT) and distractors (D) in the three experimental conditions**. Average values were computed per subject and displayed as box plots. Black diamonds indicate the respective mean over participants, red lines the median, blue boxes the 25th and 75th percentiles and whiskers the range—excluding outlier participants that are marked by red plus signs.

The fixation durations of the two or respectively three types of items differed significantly from each other in all experimental conditions [cf. Figure 8; one-way repeated measures analyses of variance; F: *F*_(1, 15)_ = 28.9, P: *F*_(1, 15)_ = 14.6, M: *F*_(2, 30)_ = 17.3; *p* ≤ 0.01 respectively, Bonferroni corrected for the three comparisons]. On average, distractor items (D) were fixated shorter than target items (PT and FT).

**Figure 9:**
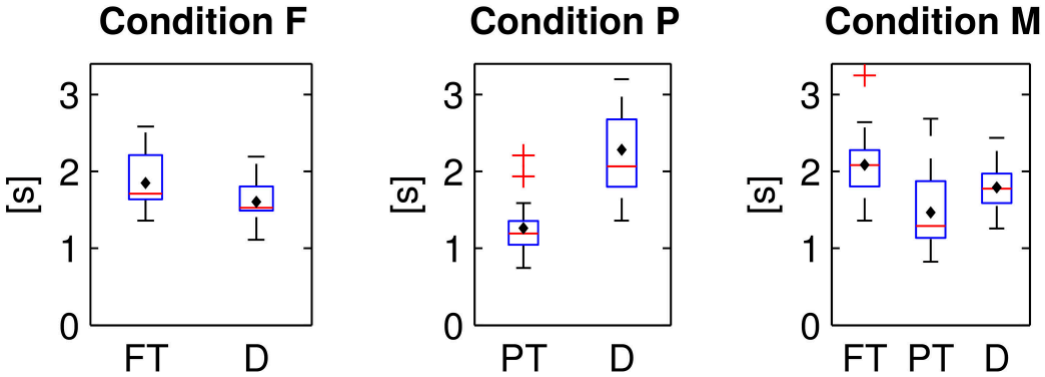
**Latencies between first appearance and first fixation of foveal (FT) and peripheral targets (PT) and distractors (D) in the three experimental conditions**.

The items were dynamically disclosed on the screen and could be subsequently fixated (cf. Section 2.1). The latencies between the first appearances and the first fixations of the two or respectively three types of items differed significantly in all conditions [cf. Figure 9, one-way repeated measures analyses of variance, condition F: *F*_(1, 15)_ = 29.3, *p* ≤ 0.01, condition P: *F*_(1, 15)_ = 76.5, *p* ≤ 0.01, condition M: *F*_(2, 30)_ = 12.6, *p* ≤ 0.01, Bonferroni corrected for the three comparisons]. On average, peripheral (PT) targets were fixated with a shorter latency after the appearance than distractors (D) and than foveal targets (FT).

The average fixation frequency of each item type in each experimental condition is listed in Table 3. Fixation frequency refers here to the number of fixations on each item type in comparison to the total number of fixations on all item types. If each single item was visited with the same probability, the fixation frequency would be 0.75 for distractors and 0.25 for targets (0.25 in the conditions F and P and 2^*^0.125 in the mixed condition M, cf. Section 2.1). Yet, the fixation frequency differed significantly from this chance level in all three conditions (two-tailed Wilcoxon signed-rank tests, *p* ≤ 0.01, Bonferroni corrected for the seven comparisons). However, the effect in terms of the difference between mean value and chance level was relatively large in condition P and comparably small in condition F and M. In condition P, more peripheral targets and less distractors were fixated than what could be expected by chance, in contrast to the conditions F and M, where the fixation frequencies reflect approximately the ratio of presented foveal targets to distractors.

**Table 3:** **Fixation frequencies averaged over participants for foveal (FT) and peripheral targets (PT) and distractors (D) in the three experimental conditions**.

	**FT**	**PT**	**D**
Condition F	0.289		0.711
Condition P		0.404	0.596
Condition M	0.143	0.141	0.716

The duration and distance of the first saccades toward foveal (FT) vs. peripheral targets (PT) vs. distractors (D) differed significantly in the conditions F and P but not in M (cf. Table 4). The duration and distance of the respective following saccades starting at the three item types (FT/PT/D) differed significantly in the conditions F and M but not in P. The statistics were calculated with one-way repeated measures analyses of variance and Bonferroni corrected for the three (F, P, and M) tests each.

**Table 4:** **Average duration in milliseconds (a) and distance in pixels (b) of the saccades toward foveal (FT) and peripheral targets (PT) and distractors (D)**.

	**Condition**	**FT**	**PT**	**D**	***F***	***df***	***p***
(a)	F	47.5		48.2	16.3	(1, 15)	≤ 0.01
	P		47.2	50.3	52.7	(1, 15)	≤ 0.01
	M	47.5	47.5	47.9	1.38	(2, 30)	>0.01
(b)	F	189		197	24.5	(1, 15)	≤ 0.01
	P		178	208	27.4	(1, 15)	≤ 0.01
	M	189	189	194	1.87	(2, 30)	>0.01
(c)	F	46.6		48.3	15.3	(1, 15)	≤ 0.01
	P		49.5	50.4	4.48	(1, 15)	>0.01
	M	46.3	46.5	48.1	8.04	(2, 30)	≤ 0.01
(d)	F	179		198	23.4	(1, 15)	≤ 0.01
	P		200	210	3.71	(1, 15)	>0.01
	M	177	178	198	17.5	(2, 30)	≤ 0.01

The duration (c) and distance (d) of the respective following saccades are given below. The results of the statistical comparisons between FT vs. PT vs. D are listed in the columns F, df and p (one-way repeated measures analyses of variance).

## 4. Discussion

### 4.1. Search task performance

The comparably large percentages of correct responses and the small absolute differences between response and true number of targets document that the participants were able to complete the task. The task performance was better in the experimental condition P than in condition F, where the targets were less salient and apparently missed more likely. The result of the mixed condition M, where both types of target items were presented, was situated in between the results of P and F (cf. Section 3.1).

### 4.2. Target estimation with EEG and eye tracking features

Spatio-temporal patterns present in the neural data and gaze features as measured with the eye tracker were exploited to estimate which items displayed on the screen were relevant (targets) in this search task with unrestrained eye movements. Both EEG and eye tracking data contained information that allowed to discriminate targets and distractors in all three experimental conditions (cf. Table 1 in Section 3.2). Crucially, the classification performance was significantly better than chance also in condition M, which modeled the more realistic scenario of a mixed item saliency. Mixed saliency leads to a temporal uncertainty of the neural correlates of target recognition (cf. Section 4.3), which was probably the reason for the lower classification performance in condition M in comparison to the conditions F and P, where target items of only one type were presented respectively. The multimodal classification of EEG and eye tracking features resulted in a better performance than when either one or the other modality was used alone (cf. Section 3.2). Thus, the two modalities contain apparently complementary information for relevance estimation.

Eye movements are often avoided or at least constrained in EEG experiments because they can result in artifacts that deteriorate the data quality of EEG recordings (Plöchl et al., 2012) and/or constitute a confounding factor. In recent investigations, which were studying EEG and eye tracking in search tasks, eye movements at a slow pace were required (Kaunitz et al., 2014) or only long fixations were included (Brouwer et al., 2013) in order to avoid contaminations by eye movements during the interval of the late positive component. In a third study, the eye movements were otherwise constrained because the subjects had to press a key on the keyboard while fixating on the target, or to maintain the fixation on the target for at least 1 s (Dias et al., 2013). Yet, restricting the eye gaze would be impractical for most real applications. For this reason, our experimental setting was as close as possible to an application scenario. The subjects could look around without any constraints. In order to check if neural signals were indeed the basis of the previously presented EEG classification results, the classifications were additionally performed with features from the electrooculogram only. In this way, we could test whether the neurophysiologic results can be explained alone by the differences in the eye movements for targets and distractors, conveyed by eye artifacts to the EEG data. The EOG classification results did not exceed the chance level significantly (cf. Table 1). Hence, neural signals provided probably the information to classify between target- and distractor EEG epochs. Compare also the discussion in Section 4.3 and 4.5. Classification results using EOG and eye tracking data were presumably different because the features for the EOG classification were extracted in the same way like for the EEG classification. The fixation durations were not estimated from the EOG signal.

In the mixed condition M, visual recognition could happen both in foveal and in peripheral vision. Two additional analyses of this condition were conducted (the results are listed in Table 2):

- *Combined classifier.* Letting two classifiers learn the patterns of foveal and peripheral recognition individually, and applying them in combination, slightly improved the average performance in comparison to the standard classification (compare Table 2, first row, with Table 1, row “EEG,” column “M”). However, this improvement was not significant and therefore, it can not be stated that the combined classifier was better suited to cope with the temporal variability of neural processes related to target recognition (cf. Figure 5, column “Fixation”) than the standard classifier, which did not take the variable stimulus saliency into special consideration. Both parts of the combined classifier used features from fixation-aligned EEG epochs—even though appearance-aligned EEG epochs seem to be particularly suited for peripheral target detection (cf. Figures 4, 5). Yet, fixation-aligned features were almost equally suited for classification in the experimental condition P (Table 1, row “EEG,” column “P”) as appearance-aligned features (cf. last paragraph of Section 3.2) and fixation-aligned EEG epochs are presumably available more frequently in an application scenario, while the popping up of items in peripheral vision is rather specific for the experiment presented here.
- *Split analysis.* Either foveal or peripheral targets were classified against distractors using fixation-aligned EEG epochs from condition M only. This approach can serve as upper bound reference of what could be achievable, if we knew whether a target can be recognized in peripheral vision or not. This knowledge can not be expected in a realistic setting. For foveal targets, classification performance improved in comparison to the standard analysis (compare Table 2, second row, with Table 1, row “EEG,” column “M”)—probably due to the reduced temporal variability of the neural response (cf. Figure 5, column “Fixation”). The result was comparable to the classification of fixation-aligned EEG epochs in condition F (cf. Table 1, row “EEG,” column “F”). However, classifying only peripheral targets vs. distractors did not result in an improvement in comparison to the standard analysis.

*Appearance-aligned EEG features.* In all experimental conditions, information was present in the EEG data about whether a target or a distractor item had just appeared in peripheral vision. Classification performance was presumably better in condition P than in the conditions F and M, because in the former peripheral detection was facilitated by the stimulus design. This type of prediction is relatively specific for our gaze contingent stimulus presentation (where items appeared in peripheral vision, cf. Section 2.1). In contrast, the prediction based on fixation-aligned EEG epochs can be more widely applied in a human-computer interaction setting and was, therefore, main focus of the target estimation presented in this paper. Yet, the analysis of appearance-aligned EEG epochs allowed us to check if peripheral vs. foveal target recognition was experimentally induced indeed (compare also the next chapter 4.3).

Please note that AUC-scores based on the predictions of single EEG epochs can not be directly compared with the class selection accuracies which are typically reported in the literature about brain-computer interfaces. A “Matrix-” or “Hex-O-Speller,” for instance, usually combines several sequences of several classifications for letter selection, which leads to an accumulation of evidence (cf. Figure 7 and respectively, Figure 4 in Treder and Blankertz, 2010; Acqualagna and Blankertz, 2013).

### 4.3. Characteristics of target and distractor EEG epochs

Target and distractor EEG epochs were class-wise averaged and differences between the two classes were statistically assessed in order to understand the underlying reasons for the results of the classifications and to gain insight into the neural correlates of peripheral and foveal target recognition. Characteristic patterns were present in the neural data depending on whether a target or a distractor was perceived (cf. Figures 4, 5 in Section 3.3). Their spatio-temporal dynamics suggest that the presence of the P300 component (also called P3) differed between the two classes. This component is a positive deflection of the ERP at around 300 ms (or later) after stimulus presentation and is known to be expressed more pronounced for stimuli that are being paid attention to (here: targets) than for non-relevant stimuli (here: distractors) (Picton, 1992; Polich, 2007). We could reproduce the findings of other studies with search tasks, where a late positive component, probably the P300, differed between fixations of targets and distractors (Brouwer et al., 2013; Kaunitz et al., 2014; Devillez et al., 2015).

The saliency of target discriminative information was varied in the experiment. Accordingly, target recognition could happen either immediately after item appearance in peripheral vision, or not until the item was fixated and in foveal vision, which was reflected in the neural data as follows:

- Clear differences between appearance-aligned target and distractor EEG epochs were found in condition P in contrast to condition F (cf. Figures 4, 5, column “Appearance”) because only peripheral targets could be recognized directly after their appearance in peripheral vision. The mixed condition M was designed with the objective to model the uncertainty of a more realistic setting where recognition can happen both in foveal and in peripheral vision. Here, both types of targets were presented and, consequently, a superposition was found of the effects from condition F and P.
- Peripheral targets could be recognized already before fixation onset in contrast to foveal targets. For this reason, differences between target and distractor EEG epochs were found in condition P at earlier time points, with respect to the fixation-onset, than in condition F (cf. Figures 4, 5, column “Fixation”). As it can be expected, condition M represents a mixture of the effects from condition F and P.

These findings match the results of the classifications with spatial features, which had the objective to learn how the neural correlate of target recognition evolves over time after item appearance or fixation, while exploiting the multivariate nature of the multichannel EEG data (cf. Figure 6).

Mid-line electrodes, mainly at central, parietal and occipital positions, were most discriminative (cf. Figures 5, 7). Hence, the results indicate that classification is not based on eye movements or facial muscle activity. These would cause higher classification performances in channels at outer positions, which are not observed here.

For Figure 5, the EEG signals were analyzed independently for all electrodes and time points. The resulting multiple testing problem was addressed with Bonferroni correction. Even though this correction is a rather conservative remedy (considering the large number of electrodes and time points), it was suited to show that the timing of the neural responses was different between conditions. The multiple testing problem could be avoided, e.g., with a general linear model with threshold free cluster enhancement (cf. Ehinger et al., 2015).

### 4.4. Eye gaze characteristics

Targets were fixated longer than distractors (cf. Figure 8) and saccades to/from targets were quicker and shorter then those to/from distractors (cf. Table 4). Apparently, target prediction based on eye tracking features (cf. Table 1) was therefore possible. The longer fixation duration for targets was presumably caused by the task, because the count had to be increased by one upon the detection of a target in contrast to the recognition of a distractor that allowed to directly pursue the search for the next target (cf. also the implications for the use case in the last paragraph of Section 4.6).

The results of the eye movement analysis demonstrate that the experimental conditions effectively induced the intended effect of peripheral vs. foveal target detection for the following reasons:

- Peripheral targets were fixated earlier after their first appearance than foveal targets and distractors—probably because they could be recognized as targets already in peripheral vision (cf. Figure 9). Besides, the saccades to peripheral targets were quicker and shorter than to distractors (cf. Section 3.4).
- The increased fixation frequency of peripheral targets in condition P (cf. Table 3) suggests that peripheral targets could be discriminated from distractors indeed in peripheral vision. Apparently, target detection in peripheral vision resulted in saccades to targets while leaving aside distractors. In contrast, fixation frequencies almost equaled the actual percentages of targets and distractors in condition F and M. In those conditions, each item had to be fixated to determine whether it is a target (the small but significant differences between the mean fixation frequencies and the chance levels were presumably caused by the rule to early stop a repetition as soon as all targets had been fixated, cf. Section 2.1).

### 4.5. Interference of eye movements with the EEG

We suggest that the classification with EEG data (cf. Section 4.2) was successful because a late positive component, evoked by cognitive processes, differed between targets and distractors (cf. Section 4.3). However, the hypothesis can be proposed that not cognitive processes but eye movements were responsible for the classification results. The fixation durations were shorter than the EEG epochs and shorter for targets than for distractors (cf. Sections 4.4 and 2.4.2). Accordingly, the following saccade occurred still during the EEG epoch and at earlier time points in the case of targets in comparison to distractors. Saccades can interfere with the EEG because the eye is a dipole, due to activity of the eye muscles and via neural processes in the visual or motor cortex: the presaccadic spike affects the EEG signal immediately before the saccade and the lambda wave about 100 ms after the end of the saccade—both resulting in a positive deflection in particular at parietal and respectively, parieto-occipital electrodes (Blinn, 1955; Thickbroom and Mastaglia, 1985; Thickbroom et al., 1991; Dimigen et al., 2011; Plöchl et al., 2012). We can not avoid this interference in unconstrained viewing.

Nevertheless, potentials related to cognitive processes were likely the predominant factor for the classification results and not potentials related to the following saccade for the reasons set out below: the time shift of the discriminative information (in fixation-aligned EEG epochs) between the experimental conditions F and P (cf. Figure 5, right column) can not be explained by differences in the eye movements because the fixation durations in F and P were similar (cf. Figure 8). A cognitive EEG component (such as the P300) is a more likely reason for the time shift because recognition was possible in condition P in peripheral vision, i.e., before fixation-onset, but only after fixation-onset in condition F.

In order to examine if the found difference patterns (cf. Figure 5, right column) are related to a cognitive EEG component and to assure that they were not caused by the following saccade, a further test was performed and EEG epochs were selected with a corresponding fixation duration longer than 500 ms. The resulting difference patterns between target and distractor EEG epochs were similar to the case where the fixation duration was less or equal than 500 ms and to the case where all EEG epochs were used. The differences appeared again before 500 ms and thus before the following saccade (cf. Supplementary Figure).

Furthermore, if presaccadic spike and lambda wave were indeed responsible for the difference between target and distractor EEG epochs, we would expect a discriminative pattern, which was not observed here (cf. Figure 5): the next saccade is expected on average 260 ms after fixation-onset for distractors and after 440 ms in the case of targets (cf. Section 3.4 with Figure 8). The presaccadic spikes can be assumed to occur just before these time points and the corresponding lambda waves about 150 ms later (including 50 ms for the duration of the following saccade). Both presaccadic spike and lambda wave are known to result in a parietal positivity (see above). Accordingly, the difference of target minus distractor related potentials is expected to be negative roughly at around 260 ms (distractor spike) and 410 ms (distractor λ) accompanied by a positivity at around 440 ms (target spike) and 590 ms (target λ). However, such a pattern was not observed (signed *r*^2^-values in Figure 5, column “Fixation”). Instead, a P300-like pattern was predominant, which occured earlier when target recognition in peripheral vision was possible than when foveal vision was necessary (cf. Section 4.3).

Moreover, we could show that EEG contains information complementary to the information from the eye tracker (cf. Section 4.2)—even if the eye tracker measures the fixation durations very accurately in contrast to the indirect measurement with EEG. Still, EEG added information—presumably because cognitive processes were captured on top of mere effects due to the eye movements. Furthermore, the results of the individual classifications with EEG features were not correlated with the results using eye tracking features. Finally, the classification of feature vectors from the electrooculogram, which were extracted just like the EEG features, was not possible. The findings mentioned contradict the hypothesis that the difference in the fixation duration made an important contribution to the EEG classification. The long-term aim is relevance detection for tasks that are cognitively more demanding than the simple search task used here. Then, eye movements might not be sufficiently informative about the relevance anymore, but accessing information about cognitive processes might be required.

### 4.6. Limitations

In view of its practical application, it has to be considered that the implicit information provided by the classifier based on EEG and eye tracking data comes with a non-negligible uncertainty. The classification performance remained considerably below an AUC of 1, which does not suffice for a reliable relevance estimation of each single item after a single fixation. This issue can be overcome by adapting the design of the practical application to the uncertainty—e.g., by combining the information derived from several uncertain predictions. Persons make several saccadic eye movements per second and, thus, EEG epochs aligned to the fixation-onsets provide a rich source of data. While the information added with each single saccade might be only a small gain, the evidence about what is relevant for the user is accumulating over time. The same strategy is followed in BCI where typically several classifications are combined for class selection. Besides, the classifier could be augmented with information derived from other sources (such as peripheral physiological sensors or the history of the user's input).

The discriminability between targets and distractors based on EEG and eye tracking data may to a substantial degree depend on the particular stimuli in use. Although a step toward reality was made and constraints regarding the stimuli were relaxed, there are more parameters to be considered. In this study, the saliency of the target items was varied on two levels only and the presentation style was always the same (the items popped up and remained at the original position). Besides, the decision about whether an item was target or not was easy and of invariant difficulty. However, in real applications, a saliency continuum can be expected, the presentation style can be diverse (items can fade in or move; Ušćumlić and Blankertz, 2016) and more cognitive effort can be required to evaluate the relevance of a stimulus. Thus, even more temporal variability is expected, with corresponding implications for classification. In this context, it can be noted that the temporal variability of neural responses in “real-world” environments is a problem recently addressed in the EEG literature, albeit in other respects (Meng et al., 2012; Marathe et al., 2013, 2014).

While here only effects related to the stimulus saliency were examined, it is known that the task has a large influence on the visual attention (cf. Kollmorgen et al., 2010; Tatler et al., 2011). In this experiment, target items were task relevant because they had to be counted by the subject. However, it should be investigated in the future whether the classification algorithm learned to detect neural correlates of target recognition indeed or merely the effects of counting, which was not required for distractors. Studies tackling several of the problems mentioned in this section are in preparation.

## 5. Conclusion

It was demonstrated how EEG and eye tracking can provide information about which items displayed on the screen are relevant in a search task with unconstrained eye movements and a mixed item saliency. Interestingly, EEG and eye tracking data were found to be complementary and neural signals related to cognitive processes were apparently captured despite of the unrestricted eye gaze. Broader context of this work is the objective to enhance software applications with implicit information about what is important for the user. The specific problem addressed is that the items displayed in real applications are typically diverse. As a consequence, the saliency of target discriminative information can be variable and recognition can happen in foveal or in peripheral vision. Therefore, an uncertain timing, relative to the fixation-onset, of corresponding neural processes can be expected. The classification algorithm was able to cope with this uncertainty and target prediction was possible even in an experimental condition with mixed saliency. Accordingly, this study represents a further step for the transfer of BCI technology to human-computer interaction and in the direction of exploiting implicit information provided by physiological sensors in real applications.

## Author contributions

Conception of the study by MW, JG, and BB. Implementation and data acquisition by JG and MW. Data analysis by MW. Manuscript drafted by MW and revised by JG and BB.

## Funding

The research leading to these results has received funding from the European Union Seventh Framework Programme (FP7/2007-2013) under grant agreement n° 611570. The work of BB was additionally funded by the Bundesministerium für Bildung und Forschung under contract 01GQ0850.

### Conflict of interest statement

The authors declare that the research was conducted in the absence of any commercial or financial relationships that could be construed as a potential conflict of interest.
