# Supplementary material for: Classification of Eye Fixation Related Potentials for Variable Stimulus Saliency
Source: Front Neurosci. 2016 Feb 15;10:23. doi: 10.3389/fnins.2016.00023 (PMC4753317; doi:10.3389/fnins.2016.00023)
Supplement: Supplementary file 1 [file SupplementaryFigure.PDF]

frontiers

Supplementary Material: Classification of eye fixation related potentials for variable stimulus saliency

Markus A. Wenzel*, Jan-Eike Golenia and Benjamin Blankertz*

* markus.wenzel@tu-berlin.de, benjamin.blankertz@tu-berlin.de

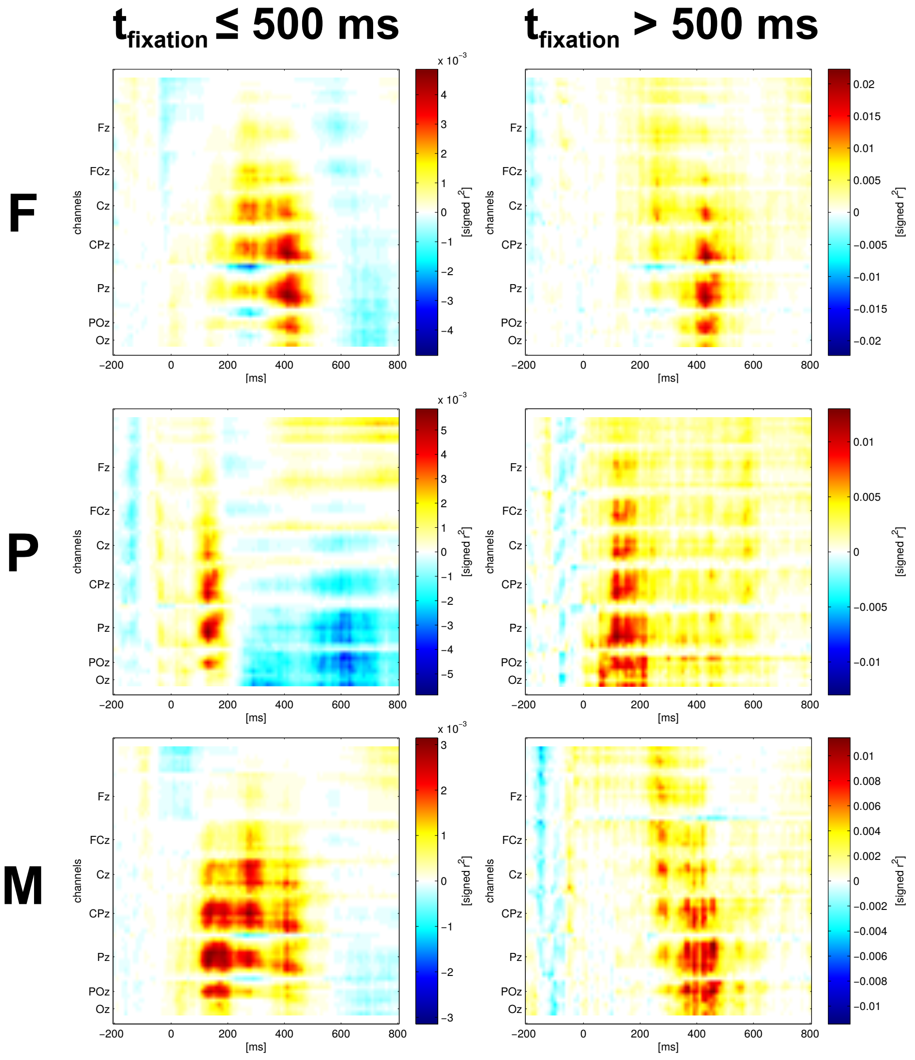

Supplementary figure. Statistical differences between target and distractor (fixation-aligned) EEG epochs with a corresponding fixation duration shorter/longer than 500 ms. The rows show the results of the experimental conditions F, P and M. Compare with figure 5, column ‘Fixation’, in the paper. Note that only a small proportion of the EEG epochs remained with the criterion of $>$ 500 ms because the items were inspected quicker in the majority of the cases.
